# Spondyloarthritis and Strength Training: A 4-Year Report

**DOI:** 10.3390/jfmk6030058

**Published:** 2021-06-24

**Authors:** Roberto Cannataro, Lorenzo Di Maio, Andrea Malorgio, Matteo Levi Micheli, Erika Cione

**Affiliations:** 1 Galascreen Laboratories, University of Calabria, 87036 Rende, Italy; 2 Department of Pharmacy, Health and Nutritional Sciences, University of Calabria, 87036 Rende, CS, Italy; 3 4MOVE Srl, 50126 Florence, Italy; lorephone@gmail.com; 4 Be Active, 56021 Cascina, PI, Italy; andrea@beactivestudio.it; 5 Department of Experimental and Clinical Medicine, University of Pisa, 56121 Pisa, Italy; 6 Department of Experimental and Clinical Medicine, University of Florence, 50100 Florence, Italy; matteo.levimicheli@unifi.it; 7 M. Marella Laboratory of Motor Sciences Applied to Medicine, 50100 Florence, Italy

**Keywords:** spondyloarthritis, strength training, deadlift, bioimpedance, high intensity, powerlifting, HIIT

## Abstract

Peripheral spondyloarthritis (SpA) has predominant peripheral (arthritis, enthesitis, or dactylitis) involvement. The severity of the symptoms can have a significant impact on the quality of life. There is no therapeutic gold standard, and physical exercise, with the opposition of resistance, remains controversial. Herein, we report the case of a woman who, at the age of 50, comes to our center with evident motor difficulties. She was previously diagnosed with SpA and was in therapy with a biological drug (adalimumab) for over one year. The training program and the nutritional intervention plan improved her condition, as pointed out by WOMAC, SQS, RAD-36 questionnaire, and BIA analysis, suspending biological therapy for almost two years. During this period, she achieved in sequence: (i) the Italian master deadlift championship, and (ii) the Italian master powerlifting championship, both for two consecutive years.

## 1. Introduction

The characteristic features of spondyloarthritis (SpA) are inflammation in the axial skeleton (sacroiliitis, spondylitis) and inflammation in the peripheral joints (arthritis) and entheses (enthesitis). According to the leading clinical symptom, SpA is differentiated in either the predominant axial or the peripheral form [1]. A discriminant for the diagnosis of SpA is the presence in serum of human leukocyte antigen (HLA)-B27 [2]. The therapy consists of physiotherapy, nonsteroidal anti-inflammatory drugs (first line), and biologicals (second line) [3]. Physical exercise is also becoming part of the therapy. However, the administration methods remain elucidated, i.e., frequency, intensity, and training volume. Under the recommendations of the American College of Sports Medicine (ACSM), it should, in any case, follow six principles: (i) overload and adaptation, (ii) progression, (iii) specificity, (iv) recovery, (v) reversibility, and (vi) individuality [4,5]. The proposed exercises are often of low intensity with the advantage of keeping the subject safe, but the effectiveness and long-term adherence to the program can be limited [6]. Recently, high intensity and/or weight training is also being applied with encouraging results [7]. We applied weight training to a case of osteoarthritis with excellent results [8]. Herein, we are presenting a patient of 51 years old (at the beginning of the program) diagnosed with axial SpA (AxSpA) aged 50 under adalimumab biological therapy (since 2016). After our intervention, the AxSpA went into remission (2018), and the biological was dismissed. At the beginning of 2020, and during the lockdown due to the COVID-19 outbreak, the recrudescence of disease came out and the biological therapy was restored. We decided to publish this case report as we aimed to support the hypothesis that strength training could be used, even in extreme cases such as this one, or knee osteoarthritis [8].

## 2. Materials and Methods

### 2.1. Clinical Characteristics and Questionnaires

The patient is a 50-year-old woman (at the beginning of the program) diagnosed with AxSpA (operated on in 2014) when she was 44 by a team of physicians of Careggi Hospital in Florence, Italy via HLA-B27 genotyping. She has been under adalimumab therapy for a year, from 2016 to 2017, and has no other concomitant pathologies. Despite the therapy, she complains of pain, difficulty sleeping and performing some daily gestures, and she comes to our observation with the help of a crutch. An assessment of pain and quality of life was carried out through the Western Ontario and McMaster Universities Arthritis Index (WOMAC) [9], Sleep Quality Scale (SQS) [10], and RAND-36 [11] questionnaires.

### 2.2. Nutritional Plans

The patient followed a nutritional plan with a protein intake of 1 g × Kg of body weight, a lipid of 0.7 g × Kg of body weight, and the remaining calorie intake from carbohydrates to reach the caloric amount to guarantee a deficit of about 200 Kcal, compared to previous habits. Much attention was paid to glycemic peaks, to avoid them being frequent and intense. They were guaranteed a daily intake of fruit, vegetables, and wholemeal starches; with additional food supplements of fish oil (DHA + EPA 50% 2 g per day), Vitamin C (500 mg twice a day), and vitamin D (2000 UI in the morning every day) provided by 4+ Nutrition Padua-Italy.

We operated a ketogenic diet for three months with less than 30 g of carbohydrates per day during the recrudescence. The caloric deficit was 250 Kcal and a ratio of 1:2 proteins: fats.

### 2.3. Bioimpedance Analysis

We applied bioimpedance analysis (BIA) [12,13,14], performed with a bioimpedance analyzer (BIA 101 Anniversary, Akern, Florence, Italy) using a phase-sensitive device with an alternating current at a frequency of 50 kHz. The accuracy of the BIA instrument was validated before each test session, following the manufacturer’s instructions. Measurements were made on a cot isolated from electrical conductors. The subjects were in the supine position with the legs (45° compared to the median line of the body) and arms (30° from the trunk) abducted. After cleansing the skin with alcohol, two electrodes were placed on the right hand and two on the right foot. Resistance (R) and reactance (Xc) parameters were divided by the standing body height in meters. Phase angle (PhA)was calculated as the arctangent of Xc/R × 180°/. A new index is calculated as LMI (Phase Angle × Height/Resistance).

### 2.4. Training Program

The training program was gradual. The subject played tennis, but with enormous effort. In the first texts, he perceived as tiring on a scale of 7 out of 10 when walking on a treadmill at a speed of 5 Km/h with a 5% gradient, therefore the first three months were exclusively dedicated to the recovery of joint mobility with very simple free body exercises. After three months, the first High-Intensity Interval Training (HIIT) circuits were introduced, but of moderate intensity, and with the gradual introduction of overloads. After almost a year, more consistent loads were introduced through kettlebells and club bells, training to strengthen the core and intensification of HIIT circuits. In mid-2017, we introduced executions of squats and deadlifts with barbells with a series of 10 repetitions and loads of 25–30 Kg. The intensity of the HIIT circuits increased to reach heart rates of 140–160 bpm, the treadmill at a speed of 6.2 Km/h, and a gradient of 7% was perceived as a warm-up. In the first months of 2018, 5 deadlift repetitions with 65 Kg were perceived as “heavy”. Starting from this period, 3 weekly bench press and deadlift workouts were performed. In mid-2018, the 1 Repetition Maximum (1 RM) of squats, bench press, and deadlifts were respectively 60, 45, and 100 Kg. Four months later they became 77.5, 50, and 115 Kg, respectively. Our focus has been on high load strength training, where HIIT has been added as a complement but not of fundamental importance.

## 3. Results

It seems that Heavy Load Strength Training could be operated for, not only in a condition such as AxSpA, but it could be effective in supporting therapy and strongly improving the quality of life.

**Figure 1:**
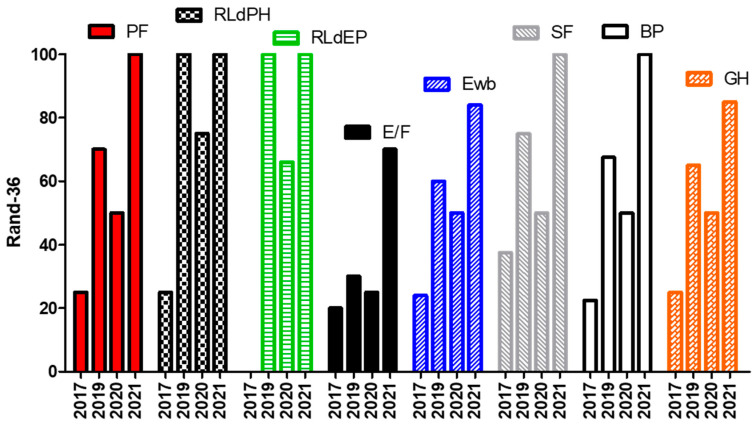
RAND-36 data recordings from 2017 to 2021. PF: Physical Functioning; RLdPH: Role Limitations due to Physical Problems; RLdPE: Role Limitations due to Emotional Problems; E/F: Energy/Fatigue; Ewb: Emotional well-being; SF: Social Functioning; BP: Body Pain; GH: General Health.

Data from WOMAC, SQS (Table 1), and RAD-36 (Figure 1) questionnaires reveal substantial improvements after two years. Data became significant compared to the last assessment operated in 2020 (Table 1 and Figure 1). In fact, in 2020, she restarted biological therapy. Aside from this, a ketogenic diet (KD) for three months (less than 30 g of carbohydrates per day, a caloric deficit of 250 Kcal, and 1:2 protein: fats). KD allowed a further improvement in weight and body composition, likely due to the anti-inflammatory and positive effects on the epigenetic status of this nutritional program [14,15].

**Figure 2:**
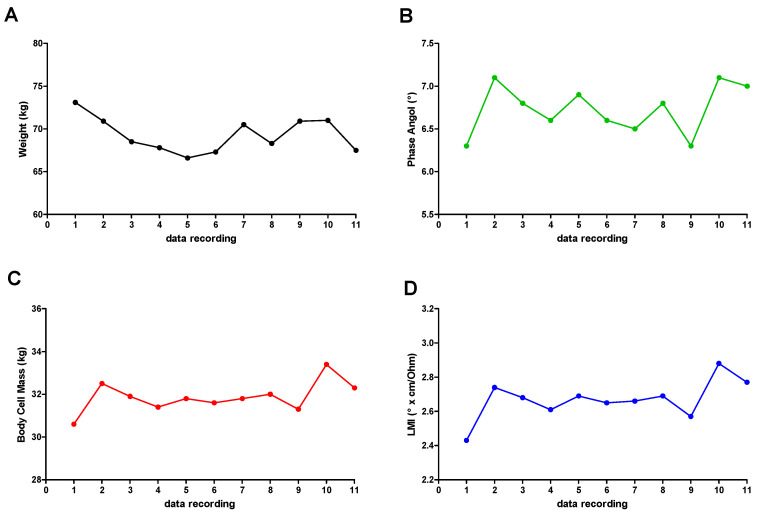
Anthropometric and BIA recording data: (**A**) weight; (**B**) PhA; (**C**) BCM; (**D**) LMI.

She lost 3.8 kg (Figure 2A), showing a clear improvement in the general condition and muscle mass. The overall improvement is evident by the phase angle (PhA) data (Figure 2B), which changed by 11%. The body cell mass (BCM) was also increased (Figure 2C) (even if this parameter is not a pure bioelectric datum) together with the new LMI, which is more closely related to muscle mass and shows an increase of 14% (Figure 2D). In Table 2, the date of BIA measurement is reported.

**Figure 3:**
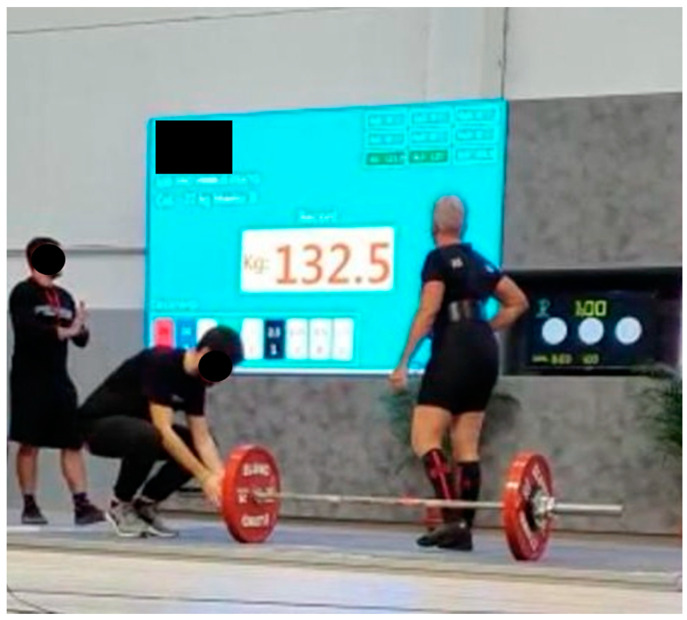
Italian deadlift championship. The AxSpA patient achieved the Italian record with 132.5 kg.

At the beginning of 2019, the first deadlift competition was performed with 120 Kg. The course reached its peak when the two victories in the Italian master championship of deadlift were obtained. For powerlifting in 2019 and 2020, the latter had the measures of 132.5 kg deadlift (Figure 3) (Italian record) and 100.5, 52.5, and 125 Kg for squat, bench press, and deadlift (Italian record), respectively.

## 4. Discussion

Axial SpA (AxSpA) is an inflammatory condition impacting the health-related quality of life. We demonstrated that weight training can be applied in AxSpA if operated scientifically and sequentially with the support of a supervised nutritional program. It could be a valid option that is normally not considered.

However, it must be emphasized that the training must be performed by qualified and trained personnel also concerning the specific pathology and operated in a highly gradual manner. The combined support of a nutritional scheme and a dietary supplement is undoubtedly desirable. Indeed, the possibility of carrying out high-intensity physical activity and competing at very high levels, even hitting the Italian championship and records, have contributed to stimulating the subject to continue along this path, thus guaranteeing optimal adherence to the program.

We excluded that the great improvement she achieved was due to pharmacological treatment because she started therapy one year before meeting us without significant improvement—she came to our center using a crutch.

We can affirm that the improvements obtained in terms of quality of life are at least in part to be ascribed to training carried out in a gradual and supervised manner. Data from WOMAC, SQS (Table 1), and RAD-36 (Figure 1) questionnaires reveal substantial improvements after two years. Data became significant compared to the last research operated in 2020 (Table 1 and Figure 1). In fact, in 2020, she restarted biological therapy. Aside from that, a ketogenic diet (KD) for three months (less than 30 g of carbohydrates per day), a caloric deficit of 250 Kcal, and 1:2 protein: fats was beneficial. KD allowed a further improvement in weight and body composition, likely due to the anti-inflammatory and positive effects on the epigenetic status of this nutritional program [14,15].

The improvements are reported from all points of view, from the physical and daily activities, and a clear improvement in the mood condition and the perception of the pathology, so much so that even the resurgence following resumption of drug therapy is lived with tranquility. The multidisciplinary intervention allowed the subject, after three years, to achieve in sequence: (i) the Italian master deadlift championship, (ii) the Italian master powerlifting championship for two consecutive years (still in charge), (iii) holding the Italian category records. We want to show, at the same time, a clear improvement of the condition (WOMAC, SQS, and RAD-36 questionnaire and BIA analysis) and suspension of drug therapy for almost two years. She was holding the Italian category records for both competitions.

There are no known biomarkers of the evolution of this pathology, and the improvement is one of clinical observation rather than biochemical.

It is interesting to note how, in correspondence with the recurrence of symptoms and consequent restoration of therapy, at the end of 2020 all the bioelectrical parameters showed a marked decline. In this case, we think it was a multifactorial cause, including less (for months absent) training, poor adherence to the diet, and likely psychological factors (as stated by most people during lockdown). They returned relatively quickly to excellent values, also attributed to the nutritional program but allowing the subject to train with an athlete’s intensity. They are scheduled to compete again as soon as the COVID-19 restrictions allow it.

From the perspective closely related to the pathology, we hypothesize that the nutritional scheme and dietary supplementation contributed to limiting the inflammation due to AxSpA. In contrast, the growth factors stimulated by high-intensity training contributed to guarantee better stability and tropism of the joints [16,17].

We want to emphasize that she did not use anything additional than reported supplements. We work with professional athletes and so are aware of any substance taken voluntarily or not, even if the overwhelmed result could induce this misthinking.

## 5. Conclusions

Inflammatory spondyloarthritis such as the axial one has a significant impact on health-related quality of life. However, it is interesting to note in this case report, an improvement in both the physiological and psychological aspects; it would be interesting to replicate the training scheme on a larger sample using teamwork, as seems to be a trend in every medical field. We want to emphasize that this result cannot be seen as standard, most likely, we believe that despite the pathological condition, the subject was well predisposed, both from the physical point of view (bone structure, joint levers) and from the psychological point of view (determination and pleasure in competition), ultimately contributing to her good results. What we want to conclude is that strength training (even with high loads but proposed in a progressive and supervised way) can be considered as a valid alternative for every subject. Diet and nutritional supplementation are often applied (not always the case) but strength training is not and is often discouraged. We show the alternative that it is possible. We are wary to definitively state that strength training is the key, but it could be a valid option.

## Figures and Tables

**Table 1:** Western Ontario and McMaster Universities Arthritis Index and Sleep Quality questionnaires.

WOMAC	Pain	Stiffness	Impact on Quality of Life	Sleep Quality
T_0_ 2017	18	8	60	64
T_1_ 2019	6	3	26	40
T_2_ 2020	10	5	35	45
T_3_ 2021	1	1	4	15
% Reduction T_0_ vs. T_3_	−95	−87.5	−93.3	−76.6

**Table 2:** Date of BIA measurement.

Data Recording	
**1**	14/04/2017
2	10/05/2017
3	12/06/2017
4	13/07/2017
5	28/11/2017
6	21/02/2018
7	25/07/2018
8	24/01/2019
9	28/02/2020
10	16/07/2020
11	27/03/2021
